# Lateral bias in the domestic pig (*Sus scrofa*)

**DOI:** 10.1038/s41598-025-28801-0

**Published:** 2025-11-27

**Authors:** Grace A. Williams, Ramon Muns, Grace A. Carroll, Gareth Arnott, Deborah L. Wells

**Affiliations:** 1 School of Psychology, Queen’s University Belfast, Belfast, BT7 1NN Northern Ireland UK; 2 Agri-Food and Biosciences Institute, Sustainable Agri-Food Sciences Division, Large Park, Hillsborough, BT26 6DR UK; 3 School of Biological Sciences, Queen’s University Belfast, Belfast, UK

**Keywords:** Laterality, Pig, Behaviour, Test re-test reliability, Sex differences, Handedness, Neuroscience, Zoology

## Abstract

Lateralised motor behaviour can contribute to the study of animal welfare, with links to emotional processing, stress responses and personality. Lateral bias in the domestic pig, a species prone to poor welfare, has been subject to little scientific attention. This study therefore aimed to assess laterality in the form of side preferences in a population of farmed pigs, exploring for differences between the sexes and consistency in side preferences, both between measures and over time. Observations of side preference were recorded in fifty pigs across five measures: a snout use task, step-up task, detour task, tail curling and lying side. Snout use, step-up and detour side preferences were observed twice (3 and 6 weeks-of-age) to evaluate test-retest reliability. Pigs were significantly more likely to be ambilateral than side-preferent for both lying side and snout use at 6 weeks of age. By contrast, animals showed significant side-preferences at the level of the individual on the detour task at 6 weeks of age. Directional laterality index (LI) scores for snout use were positively correlated with those of the step-up task at 6 weeks of age, while strength of laterality index (ABSLI) scores for snout use at 4 weeks of age were positively correlated with the step-up task scores at 6 weeks of age. No other LI or ABSLI scores were significantly correlated. Findings pointed to good test-retest reliability, with animals demonstrating a significant positive correlation in the direction, although not strength, of their lateral biases for the snout use, step-up and detour tasks. A cluster analysis, employed to explore for individual lateralisation patterns across motor functions, revealed a leaning towards left-side preferences on animals’ combined step-up and detour laterality scores. Male and female animals showed no significant difference in either the strength or direction of their side preferences for any of the tasks. Overall, the results from this study point to individual-level lateralised behaviour in the domestic pig for one measure. Findings reveal a lack of sex differences and consistency between tasks, but show stability in pigs’ side preferences over time, at least in the short-term.

**Supplementary Information:**

The online version contains supplementary material available at 10.1038/s41598-025-28801-0.

## Introduction

Lateralised motor behaviour has been studied as an observable measure of cerebral functional asymmetry for numerous years^1,2^. The most prominent manifestation of lateralised behaviour in humans is that of handedness (i.e., the predominant use of one hand), with approximately 90% of people using their right hand most of the time^3,4^.

It is now known that hemispheric specialisation is not exclusive to humans, with most vertebrates^5^, and even some invertebrates^6^, showing functional asymmetries, e.g., paw/limb preferences. The evidence for laterality in non-human species is particularly exciting when considered from an animal welfare perspective. For example, motor laterality can give us an insight into an animal’s affective state^7,8^. Davidson and colleagues^9^ proposed the “emotional valence hypothesis”, asserting that each brain hemisphere is specialised in processing different types of emotion. The left hemisphere typically controls positive emotions and approach behaviour, while the right hemisphere controls negative emotions and withdrawal^7,10^. Studying an animal’s side preferences, which are generally associated with greater activity of the contralateral brain hemisphere^11^, can therefore provide a valuable insight into the emotional state of an individual, allowing us to determine whether or not it is finding any particular environment or situation stressful. Dogs, for example, generally wag their tails more to the right when looking at stimuli with a positive emotional valence (e.g., their owners), and more to the left when presented with negative emotional stimuli, e.g., unfamiliar dog^12^. In a similar vein, primates, such as baboons, have been shown to display a left-sided bias in vocal screeching in response to aggression^13^.

Lateralised behaviour may also offer insights into the personality of an animal (for reviews see^10,14^. Individuals differ in the degree to which they use one hemisphere over the other, leading to differences in behavioural responses to environmental stimuli^15^. These responses manifest themselves through consistent behavioural differences^16^, also referred to as personality^17^. Right-sided and strongly lateralised individuals are typically more explorative, sociable and/or bolder than left-sided and/or weakly lateralised individuals (for review see^14^. For example, strongly lateralised dogs have been found to be less reactive to the sounds of thunderstorms than animals without a significant paw preference^18^, while horses assessed as right-hemisphere dominant have been shown to be more fearful when presented with an unfamiliar stimulus than their left-hemisphere dominant counterparts^19^. Establishing which side of the body an animal uses, and the strength of this bias, can therefore contribute to our understanding of an animal’s temperament and corresponding welfare risk.

Lateral biases have been studied from a welfare perspective in a range of domesticated species, including dogs and cats (for reviews see^20–22^, horses^23,24^, sheep^25,26^ and cattle^27,28^. Surprisingly little attention, however, has been devoted to the study of laterality in the domestic pig, an intensively farmed species^29,30^. The limited evidence available, however, points to the presence of lateralisation^31–34^. Goursot and colleagues^33^, for example, found that 5-7-week-old male pigs show motor bias at the level of the individual, with links to personality^34^. Specifically, in line with research on other species (for review see^8^, these studies found that the left hemisphere of the brain plays a role in positive appraisal, with right-biased pigs being bolder, more explorative and more sociable than left-biased animals^34^. This research, however, was only conducted on male animals, at one point in time. The expression of laterality, however, is not stable throughout the lifespan^35^, and indeed numerous factors (e.g., age, sex, task complexity, breed, nutrition) are associated with individual side preferences^20^.

For example, some (although not all) studies have found a population split in directional laterality, with male animals of several species (e.g., primates^36–38^; horses^39,40^; dogs^41–43^; cats^44–47^ showing a preference for left limb use (right hemisphere dominance) and females leaning more heavily towards right limb bias (left hemisphere dominance). Several theories have been put forward to explain these sex differences, e.g., differential exposure to gonadal steroid hormones, notably testosterone (for review see^48^, differences in asymmetric brain organisation^49^, genetic factors (e.g., an X-linked recessive gene that leads to suppression of an autosomal right gene^50^. Given the discrepancy in findings in this area, it is important to continue exploring for lateralised sex effects in animals, particularly in species that have been overlooked in this respect. During contest behaviour, female pigs have been shown to look at conspecifics more with their right eye, while males rely more on their left eye^31^; one might therefore expect a leaning towards sex differences in directional sidedness in pigs that are in keeping with the results reported above on other animals.

The demands of the task may also have a role to play in determining what limb is used by an animal. Studies on primates and domestic chicks (for reviews see^11,16^ have shown that temporal sequencing and non-spatial tasks result in more dominant left hemisphere processing and a subsequent leaning towards right limb motor use, while spatial exercises and tasks demanding attention to a novel stimulus encourage predominately right hemisphere processing and left limb output. It is therefore important to assess side preferences using a range of tasks. Since laterality can change with age^47,51^, it is also important to explore for repeatability, assessing animals on the same measures on more than one occasion^52^. Categorising an animal as ‘left-limbed’, for example, on the basis of its performance on one test at just one point in time could provide misleading information on the emotional vulnerability of that individual if side preferences are task-specific and another measure, or testing at another point in time, might lead to the same animal being classified as ‘right-limbed’ or ambilateral. Determining whether individuals show consistent side preferences is also important for establishing the responses of animals to environmental changes (positive [e.g. enrichments] or negative).

In light of the above, the following paper aimed to assess laterality in the form of side preferences in the domestic pig, exploring for differences between the sexes and consistency in side preferences, both between measures and over time. A number of measures were used to explore for evidence of sidedness, including expressions of naturalistic behaviour (*lying side*), performance on a range of well-established tasks (*snout use*,* detour*,* step-up*)^33,44,52–54^ and tail curling. It was hoped that the study would further our understanding of laterality in an understudied species and determine which measures harbour the most value (i.e., are reliable, repeatable, easy to use) in the context of the working farm.

## Methods

### Animals and housing

**Fig. 1:**
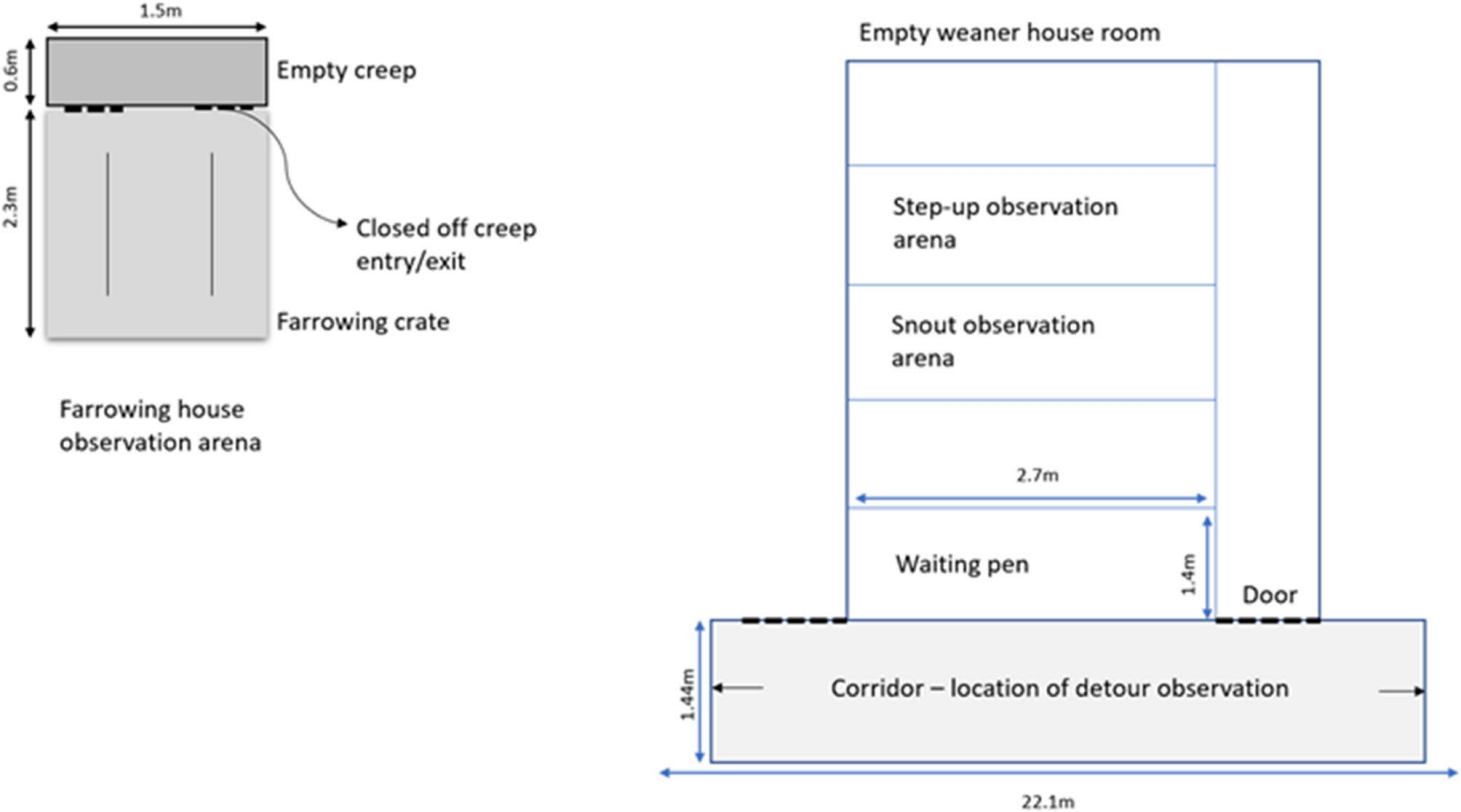
Layout of observation areas in the farrowing and weaner houses.

Fifty (26 males, 24 females) crossbred [(Landrace x Large White) x Duroc], pre-pubertal, uncastrated piglets were recruited as subjects. All pigs were housed at the Agri-Food and Biosciences Institute (AFBI) Pig Research farm in Hillsborough, Co. Antrim, Northern Ireland. The study was carried out across three consecutive replicates. Two focal litters were used in each replicate, with eight to nine pigs, balanced by sex, selected as subjects from each litter. All piglets were housed in a conventional farrowing pen (2.3 × 1.5 m) with the sow and their litter until they were weaned at 4 weeks of age. Each farrowing pen was equipped with an enclosed creep area at the front (1.5 × 0.6 m). An empty farrowing crate and its attached empty creep area were used as the observation arena for piglets studied at this age (see Fig. 1). At weaning, the two focal litters were mixed among themselves, based on their weight, to produce two groups of 8–9 pigs which were then housed across two weaner pens (≥ 0.38 m^2^ per pig) in the weaner house. Weaner pens had plastic slatted floors and were enriched with a suspended wooden log. Food and water were available *ad libitum*. Behavioural observations for animals at this age occurred in an empty weaner room, comprising five empty weaner pens (13.5 × 7.0 m). As part of routine husbandry procedures, animals had ~ 50% of their tails docked within 24 h of birth.

### Procedure

**Fig. 2:**
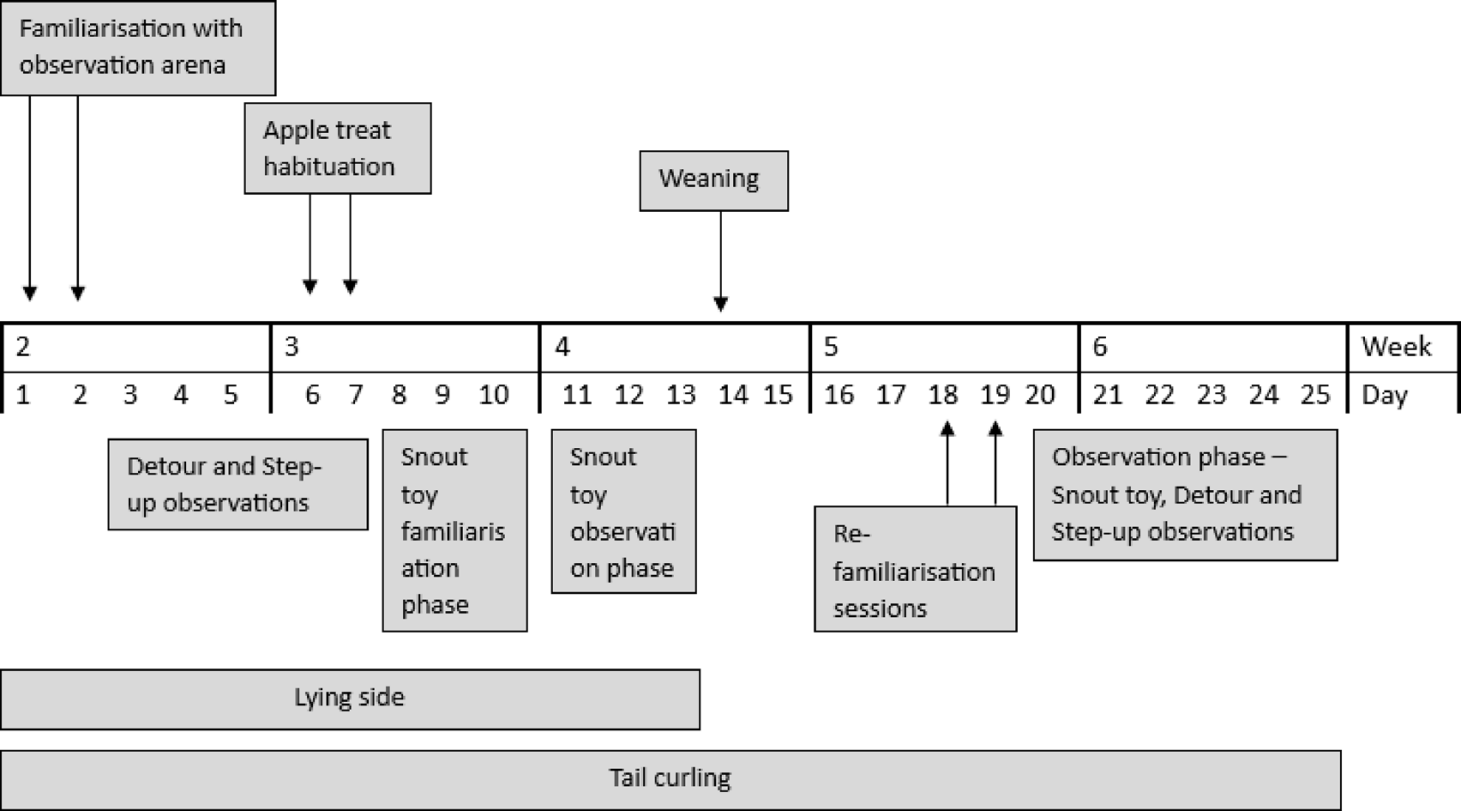
Timeline of observations.

Subject animals were recruited, and the observation period commenced on the first day of the second week of each piglet’s life (day 1 of the investigation). Days 1 and 2 of the study involved habituating the animals to handling by the experimenter (GW) and familiarisation with the observation area. Animals were brought to the observation arena in the farrowing house via a weaner trolley used as part of routine husbandry practices. The pigs had up to three 10-minute sessions in pairs to encourage familiarisation with the empty farrowing crate. A timeline of observations for the five laterality measures is presented in Fig. 2. In brief, piglets took part in the *detour* and *step-up* tasks between days 3–7 of the study. On days 6 and 7, after piglets were introduced to solid food as per normal practice (i.e., were offered creep feed inside the farrowing pen), they were habituated to the apple treat used in the tasks; two handfuls of apple pieces were distributed to each pen of animals. The familiarisation phase to the *snout use* toy took place between days 8–10 of the study. The pigs had three 10-minute paired sessions in the empty creep area to facilitate learning of the apparatus. In two of these sessions, animals had free access to the centrally positioned treat before the brick was added. They then completed two seven-minute individual sessions to habituate them to completing the observation alone. The observation phase for this measure then took place between days 11–13 of the study. *Lying side* was recorded between days 1–13 of the study, while *tail curling* measurements were recorded between days 1–25. The piglets were weaned on day 14 of the study (day 28 of life), as per standard practice. On days 18 and 19 of the study, there were two re-familiarisation sessions in the weaner house, one in pairs and one individually, to adjust the piglets to the setup of the laterality observations in this location. Animals were guided along the floor from their home pen to the observation arena in the weaner house. The second round of observations of the *snout use*,* detour*, and *step-up* tasks took place between days 20–25. *Snout use* and *detour* observations were carried out individually. The *step-up* task was completed in pairs to facilitate efficient engagement with the apparatus.

### Lateral bias measures

Five lateral bias measures were recorded for each subject animal.

#### Snout use

**Fig. 3:**
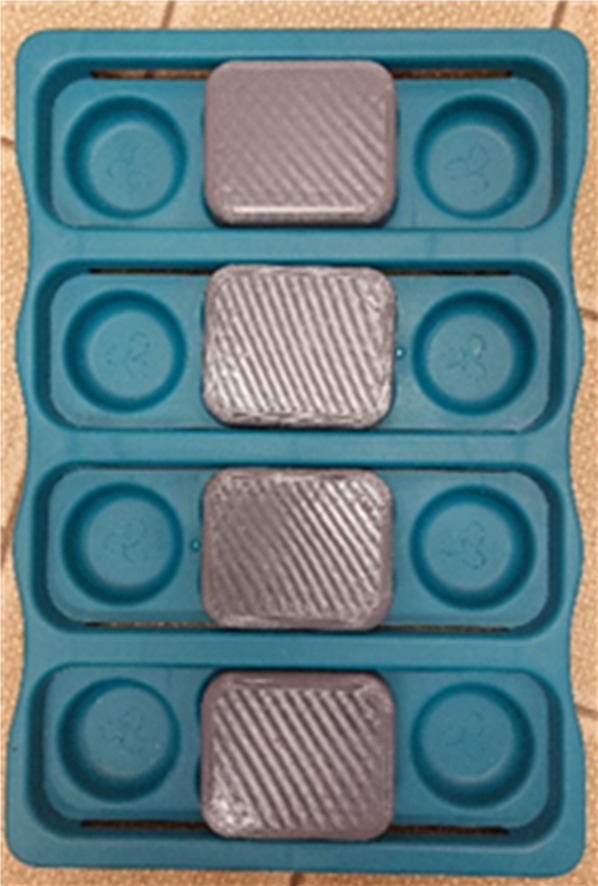
Dog brick toy used to assess pigs’ snout use laterality.

A modified ‘dog brick toy’ (Nina Ottosson by Outward Hound, Sweden, Fig. 3) was used to measure the pigs’ snout use, a method modified from that employed by Goursot et al.^33^. The toy had four plastic sliding ‘bricks’ (7 × 6 cm), under which a food treat (chopped apple) was placed. To retrieve the treat, the brick was required to be moved to the left or right. Gate barriers were positioned on either side of the toy to ensure the pig approached the apparatus centrally. The experimenter stood centrally to the toy outside the pen while the pig was undertaking the task. Treats were reloaded in the event of an animal eating all of the food. The direction in which the brick was moved (left or right) by the pig in an effort to reach the food underneath was recorded for each animal. A trial ended when the piglet successfully moved all four bricks (regardless of whether or not a pig was successful in retrieving the treat) or five minutes had elapsed. Up to eight trials were undertaken per day, with an interval of at least half an hour between trials, until 10 observations were observed for each animal. Since one of the goals of the study was to explore for consistency in side preferences over time, data were collected from each animal at two stages – 4 weeks of age in the farrowing house and 6 weeks of age in the weaner house.

#### Detour task

**Fig. 4:**
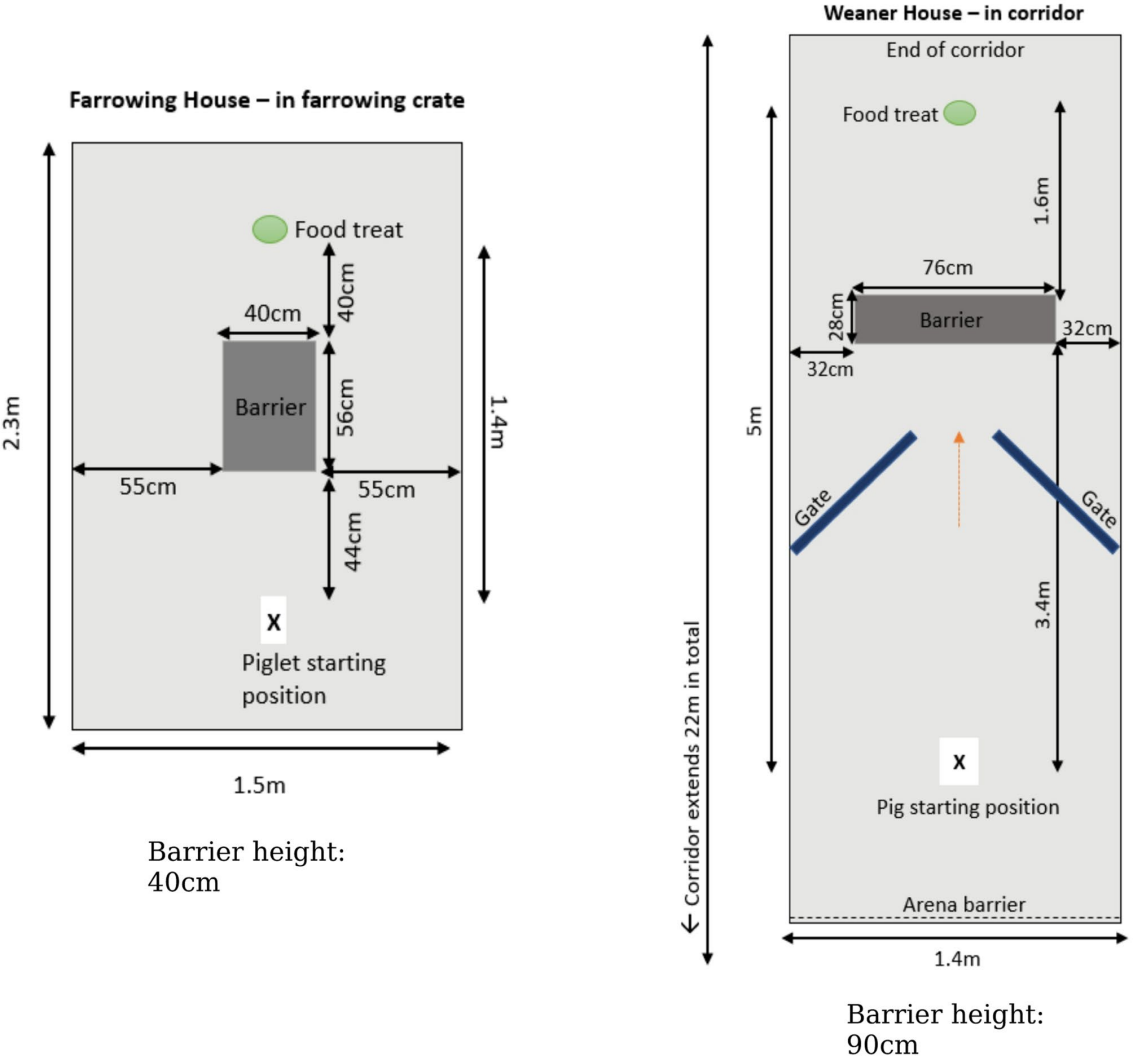
Detour task layout in the farrowing and weaner houses.

A detour task was used to determine which side pigs moved around. To familiarise animals with the setup, piglets were initially provided with a food treat (apple pieces hidden under newspaper) presented ~ 1.4 m away from a starting point in the farrowing house and ~ 5 m away in the weaner house. Animals were allowed free access to the food treat on 3 separate occasions, enabling them to habituate to being tested alone. During the observation phase, animals were presented with further opportunity to reach the food, but this time a barrier (a box in the farrowing house and an empty feeder in the weaner house) was placed in front of the food (at a distance of ~ 40 cm in the farrowing house and ~ 160 cm in the weaner house) at the midline of the pig (see Fig. 4). Starting from a central position, the direction taken by the animal to detour the barrier (left/right) was recorded for each trial. In the weaner house, gates were positioned on either side of the corridor to facilitate pigs approaching the barrier centrally. The experimenter stood behind the starting position, central to the barrier, whilst the animal completed the task. Up to 8 trials were undertaken per day, with an interval of at least half an hour between trials, until 10 observations per animal were recorded. Data were collected at both 3 and 6 weeks of age.

#### Step-up

**Fig. 5:**
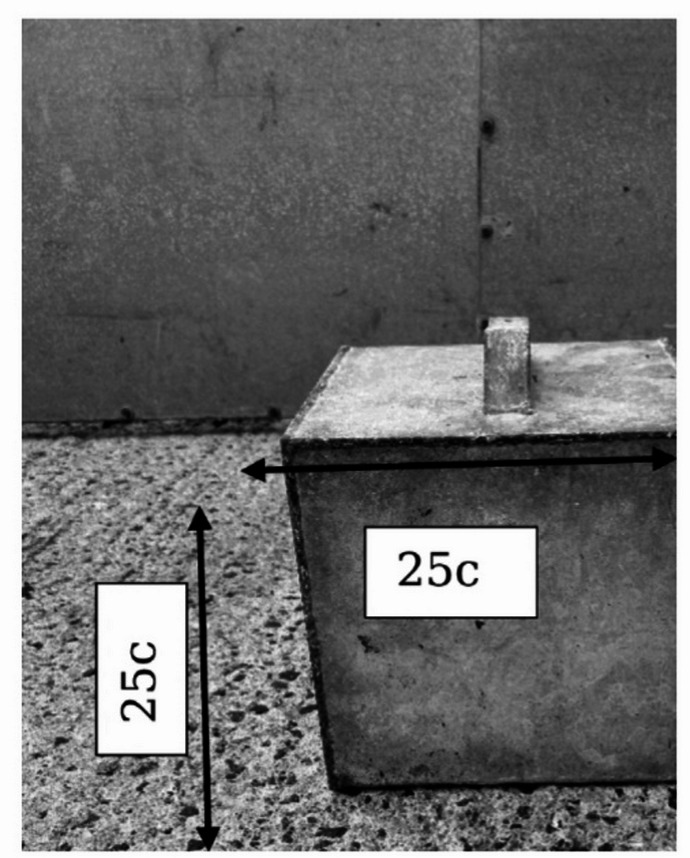
Iron block used to assess pigs’ step-up side preferences.

A step-up task, modified from a similar exercise adopted by Goursot et al.^33^, was developed for use in this study. A galvanised iron block (25 cm x 25 cm x 25 cm) was employed as the platform onto which pigs were required to step up to with their front trotters (see Fig. 5). This was placed in the centre of the empty farrowing crate in the farrowing house and in the centre of an empty pen in the weaner house. Pieces of apple, peanut butter and newspaper were placed centrally on the top of the platform to encourage the animal to step up. The animal had to reach with its front leg to perform this task; this movement helped to ensure the pig started from a stationary position. The experimenter stood centrally to the iron block outside the pen while the pig completed the task. The front leg (left or right) used by the pig to first step up onto the platform was recorded. Side preferences were only recorded when the pig started with all four legs on the floor and was completing the step-up action as a single movement (rather than as part of a run or as part of a continuing movement from the previous step-up). Up to 5 trials (each lasting 5 min) were undertaken per day, with an interval of at least half an hour between trials, until 10 observations per animal were observed. Again, to allow for an exploration of consistency in side preferences over time, observations were made at both 3 and 6 weeks of age.

#### Tail curling

**Fig. 6:**
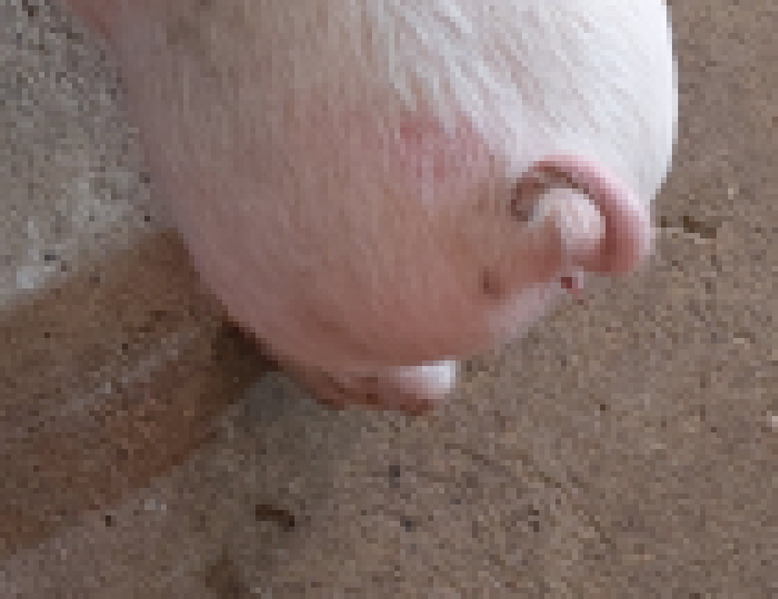
Tail curling in a piglet.

Tail curling direction was also recorded as an indicator of sidedness. The animals in this study had 50% of their tail length docked, although it was still possible to determine whether the base of the tail veered to the left or right side of the animal’s body. The protocol employed by Goursot et al.^33^ was used for reference, however, in the current investigation, tail curling observations were made from outside, as opposed to inside, the animals’ pens. Animals were required to be either walking or standing at the time of observation so that tail curling was observable to the experimenter. Direction was recorded as either left (i.e., end of tail is located to the left of the pig’s body) or right (i.e., end of tail is located to the right of the pig’s body) (Figure 6). Unlike the previous measures, tail curling was recorded at only one point in time (between 2 and 6 weeks of age). This decision reflected practical and time constraints during the farrowing house stage, where suitable opportunities for undisturbed home pen observations were limited, and pigs were not always witnessed standing during observation periods. Consequently, data collection for tail curling was extended up to six weeks of age across the duration of the study, resulting in a single complete set of 10 tail curling observations per pig.

#### Lying side

**Fig. 7:**
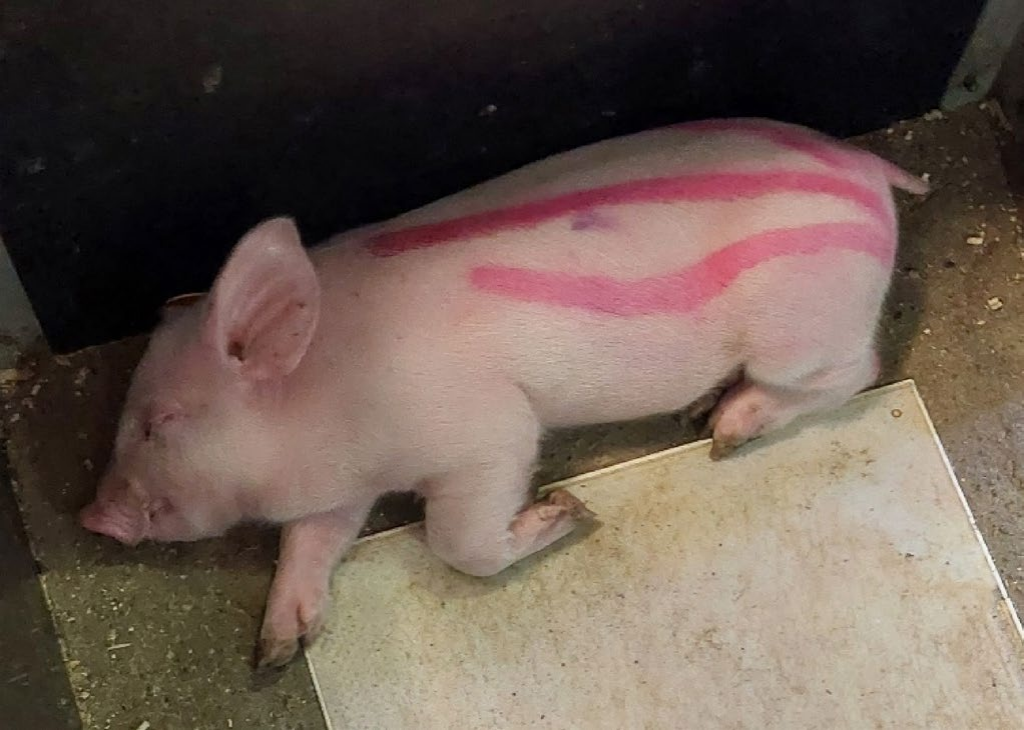
Lying side in a piglet.

Lying side was used as a naturalistic expression of side preference. Data were collected by the experimenter, who stood outside the animal’s home pen, when the subject individual was not surrounded by other lying piglets, and had adequate surrounding space to make a lying side choice (recorded as left or right, Fig. 7). Lying side was observed up to twice a day, with at least two hours between observation periods. Ten occurrences of lying side were noted for each piglet in the farrowing house, between 2 and 4 weeks of age. As with tail curling, this measure was only recorded at one timepoint, due to older pigs lying on top of each other, making lying side difficult to observe in the weaner house.

### Statistical analysis

All animals undertook task trials until 10 successful laterality observations were recorded for all measures. A full dataset was therefore available for all subjects. A series of analyses were carried out (below) to examine whether the pigs showed individual or population level laterality for each of the measures and explore whether there were differences in either the strength or direction of the pigs’ side preferences between tasks. Analysis also explored for sex differences in laterality, test repeatability and consistent patterns of lateral bias. SPSS version 30.00 was used to analyse the data.

#### Individual vs. population level laterality

##### Distribution of lateral bias

Binomial z-scores were calculated for each laterality measure, as per Wells et al.^52^, to determine whether a significant majority of pigs showed a side bias, i.e., whether the frequency of right or left side preferences exceeded that expected by chance. An alpha value of 0.05 was adopted for all analyses. Pigs with a *z*-score greater than + 1.96 (two-tailed) were classified as showing a left-sided preference (L), whilst those with a *z*-score less than − 1.96 were classified as displaying a right-sided preference (R). Animals with *z*-scores between + 1.96 and − 1.96 were classified as ambilateral (A). Binomial tests, correcting for multiple comparisons, were subsequently carried out at the level of the group for each laterality measure to determine if pigs were more likely to be side-preferent (either L or R) or ambilateral and to establish if side-preferent animals were more likely to favour one side of their body (L or R) than the other.

##### Direction and strength of laterality

For a continuous representation of laterality direction, a directional laterality index (LI) was calculated. This quantified each pig’s lateral bias on a continuum from strongly left-sided (+ 1) to strongly right sided (-1). The LI was calculated as per others^44,55^ by dividing the difference between the total number of left and right sided observations by their sum: (L-R)/(L + R). The strength of the pigs’ laterality was calculated for each laterality task by taking the absolute value of the LI scores (ABSLI scores). A Friedmann Anova test was carried out to compare the strength of pigs’ lateralisation between the different laterality tests at the later stage of testing (6 weeks of age).

##### Associations between measures of laterality

A series of Spearman’s rank order correlations were carried out to explore if either the pigs’ LI or ABSLI scores were correlated with each other for any of the measures.

##### Sex differences in laterality

Mann-Whitney *U* tests were conducted to determine if either the direction (LI) or strength (ABSLI) of the pigs’ side preferences differed between male and female animals.

##### Test repeatability

For those measures carried out twice (*snout use*,* detour*, *step-up*), Spearman’s rank order correlations were conducted to explore for associations in either the direction (LI) or strength (ABSLI) of the pigs’ side preferences for both their first and second attempts at the tasks.

##### Patterns of laterality

To explore for side preference patterns across the different laterality measures, a cluster analysis was employed to derive a combined laterality classification, as per Goursot et al.^33^. Only measures whose LI scores displayed a bimodal distribution, based on visual inspection of the histograms, were selected for inclusion. In RStudio, the ‘k-means’ clustering algorithm was applied to the LI scores from the ‘stats’ package in R^56^. As the k-means algorithm uses distances metrics, data were standardised to ensure that both features contributed equally to the clustering process. The Elbow Method, Silhouette Method and the Gap Statistic were employed to determine the optimal number of clusters to best represent the data^57,58^. The Silhouette width score was used to evaluate the quality of the clusters formed, alongside visual appraisal of the graphical representation of the clusters. Chi-squared analysis was subsequently carried out to examine whether pigs were more likely to be side-preferent (L or R) or ambilateral (A) across the tasks (i.e., whether they were more likely to have a consistent side preference across tasks (LL or RR), or whether their side preference differed (A – i.e., LR or RL) across tasks), while Mann-Whitney *U* tests explored for sex differences in the pigs’ mean LI and ABSLI scores.

### Ethical statement

This research was carried out in accordance with the relevant guidelines and regulations (Association for the Study of Animal Behaviour Guidelines for the Use of Animals, *Animal Behaviour*, 2006, 71, 245–253). Ethical approval for this study was granted by Queen’s University Faculty Research Ethics Committee (Ref: EPS 23_63).

## Results

### Individual vs. population level laterality

#### Distribution of side preferences

Analysis revealed no significant difference in the proportion of pigs that were ambilateral (A) or side-preferent (R + L: right- (R) and left- (L) sided) for most of the measures (Table 1). Pigs were more likely to be ambilateral than side-preferent for both *lying side* and *snout use* at 6 weeks of age. By contrast, animals were significantly more likely to be side-preferent than ambilateral on the *detour* task at 6 weeks of age. More of the pigs that showed a side preference on this task veered to the left than the right side of the barrier (*p* = 0.04, binomial test, ns after Bonferroni corrections).

**Table 1:** Distribution of pigs’ lateral biases for each laterality measure. The number of right-sided (R), left-sided (L) and ambilateral (A) pigs for each task are displayed. Binomial test results (P value) indicate difference between the number of lateralised (R + L) and ambilateral (A) pigs for each task (*significant result using Bonferroni adjusted significance threshold: *p* < 0.006).

Laterality test	*R* *N* (%)	L *N* (%)	*R* + L *N* (%)	A *N* (%)	*P*-value
Snout (4wks)	6 (12)	11 (22)	17 (34)	33 (66)	0.03
Snout (6wks)	6 (12)	8 (16)	14 (28)	36 (72)	0.003*
Step-up (3wks)	14 (28)	18 (36)	32 (64)	18 (36)	0.06
Step-up (6wks)	13 (26)	15 (30)	28 (56)	22 (44)	0.48
Detour (3wks)	18 (36)	14 (28)	32 (64)	18 (36)	0.06
Detour (6wks)	12 (24)	25 (50)	37 (74)	13 (26)	< 0.001*
Lying side	8 (16)	5 (10)	13 (26)	37 (74)	< 0.001*
Tail curling	13 (26)	20 (40)	33 (66)	17 (34)	0.03

#### Direction and strength of laterality

**Fig. 8:**
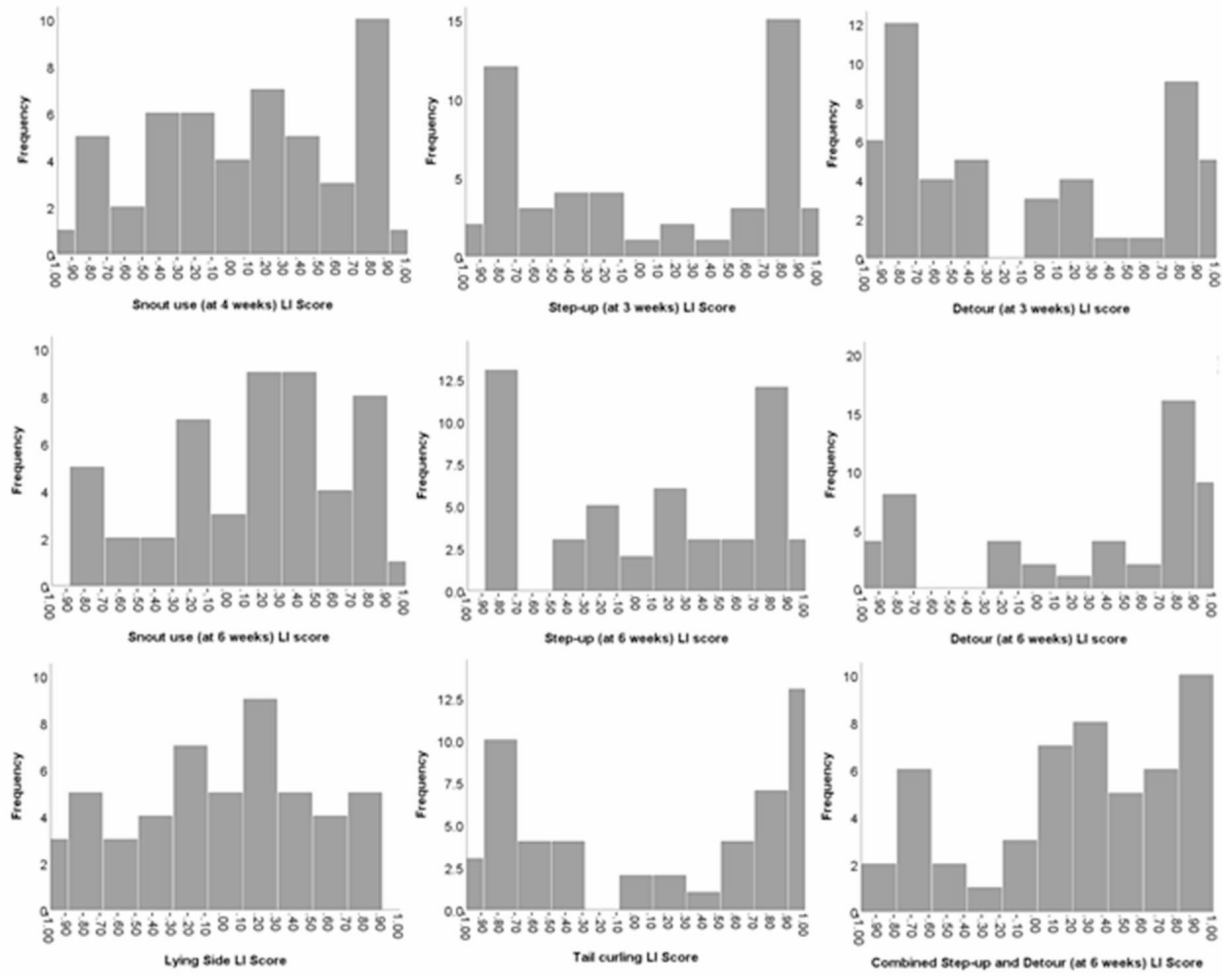
Histograms showing the directional laterality (LI) scores for laterality measures at each point in time. Each column represents a range of LI values. The height of the column shows how many pigs have LI scores within that range.

The direction of the pigs’ side preferences (LI scores), was found to follow a non-normal distribution for most measures (*p* < 0.05, Kolmogorov-Smirnov tests). Histograms for *step-up*,* detour* and *tail curling* observations showed largely bimodal characteristics (Fig. 8). Only the LI scores for *snout use* at 4 weeks of age were found to be normally distributed (*D* (50) = 0.12, *p* = 0.07), pointing towards laterality at the level of the individual, rather than the population.

A Friedmann Anova test revealed a significant difference in pigs’ ABSLI scores between the five laterality measures at 6 weeks of age (χ^2^ = 39.85, df = 4, *p* < 0.001). As can be seen from Table 2, pigs showed the highest ABSLI scores for the *tail curling* and *detour* measures. Post hoc Wilcoxon tests showed that pigs had significantly (*p* < 0.001) stronger ABSLI scores for both the *tail curling* and *detour* measures than either *lying side* (*tail curling*: Z = − 3.66; *detour*: Z = -4.05) or *snout use* (*tail curling*: Z = − 3.94; *detour*: Z = -3.95). None of the other pairwise comparisons were significant.

**Table 2:** Mean (+/-SEM) strength of laterality (ABSLI) scores for each laterality measure.

Laterality test	Mean ABSLI (±)
Tail curling	0.74 (0.04)
Detour	0.72 (0.04)
Step-up	0.59 (0.04)
Snout use	0.45 (0.04)
Lying side	0.44 (0.04)

### Associations between measures of laterality

LI scores for *snout use* at 6 weeks were positively correlated with those of the *step-up* task at 6 weeks of age (*ρ*[48] = 0.42, *p* = 0.003). None of the other LI scores were significantly associated with each other.

Analysis revealed a significant positive correlation between ABSLI scores for *snout use* at 4 weeks of age and scores on the *step-up* task at 6 weeks of age (*ρ*[48] = 0.42, *p* = 0.003). Strength of laterality scores were not significantly associated for any of the other measures.

### Sex differences in laterality

Male and female animals did not differ significantly in their LI scores for any of the measures (*p* > 0.05, Mann-Whitney *U* tests), with the exception of *tail curling* (*U* = 193.50, *p* = 0.02). Male animals were more likely to show right-sided tail curling (mean LI score = -0.08, +/-0.14), while females veered more towards left-sided tail curling (mean LI score = 0.37, +/-0.16). After correcting for multiple comparisons, however, no significant sex effects were observed for this measure.

Male and female pigs also differed significantly in their strength of laterality for *tail curling* (*U* = 417.50, *p* = 0.03). Females were more strongly lateralised (mean ABSLI score = 0.83 +/ 0.04) than males (mean ABSLI score = 0.65 +/- 0.05). After correcting for multiple comparisons, however, no significant sex effects were observed.

### Test repeatability

Results revealed a significant positive correlation in LI scores between the two timepoints for all of the tasks that were undertaken twice; *snout use* (*ρ*[48] = 0.44, *p* = 0.001), *detour* task (*ρ*[48] = 0.47, *p* < 0.001), *step-up* task (*ρ*[48] = 0.65, *p* < 0.001).

There was no significant correlation in the pigs’ ABSLI scores between the two ages for the *snout use* task (*ρ*[48] = 0.13, *p* = 0.36). ABSLI scores for the *detour* task (*ρ*[48] = 0.35, *p* = 0.01) and *step-up* task (*ρ*[48] = 0.33, *p* = 0.02) were not significantly correlated between the two timepoints after correcting for multiple comparisons.

### Patterns of laterality

Both the *step-up* and *detour* tasks had a bimodal distribution and were therefore included in a combined laterality classification. Since a bimodal distribution was evident at both ages for these tasks, and there was good test-retest reliability for both measures, only the later timepoint (6 weeks of age) was selected for inclusion in the cluster analysis. *Tail curling* also followed a bimodal distribution, however it was excluded from the combined laterality classification due to concerns with this measure (see Discussion).

**Fig. 9:**
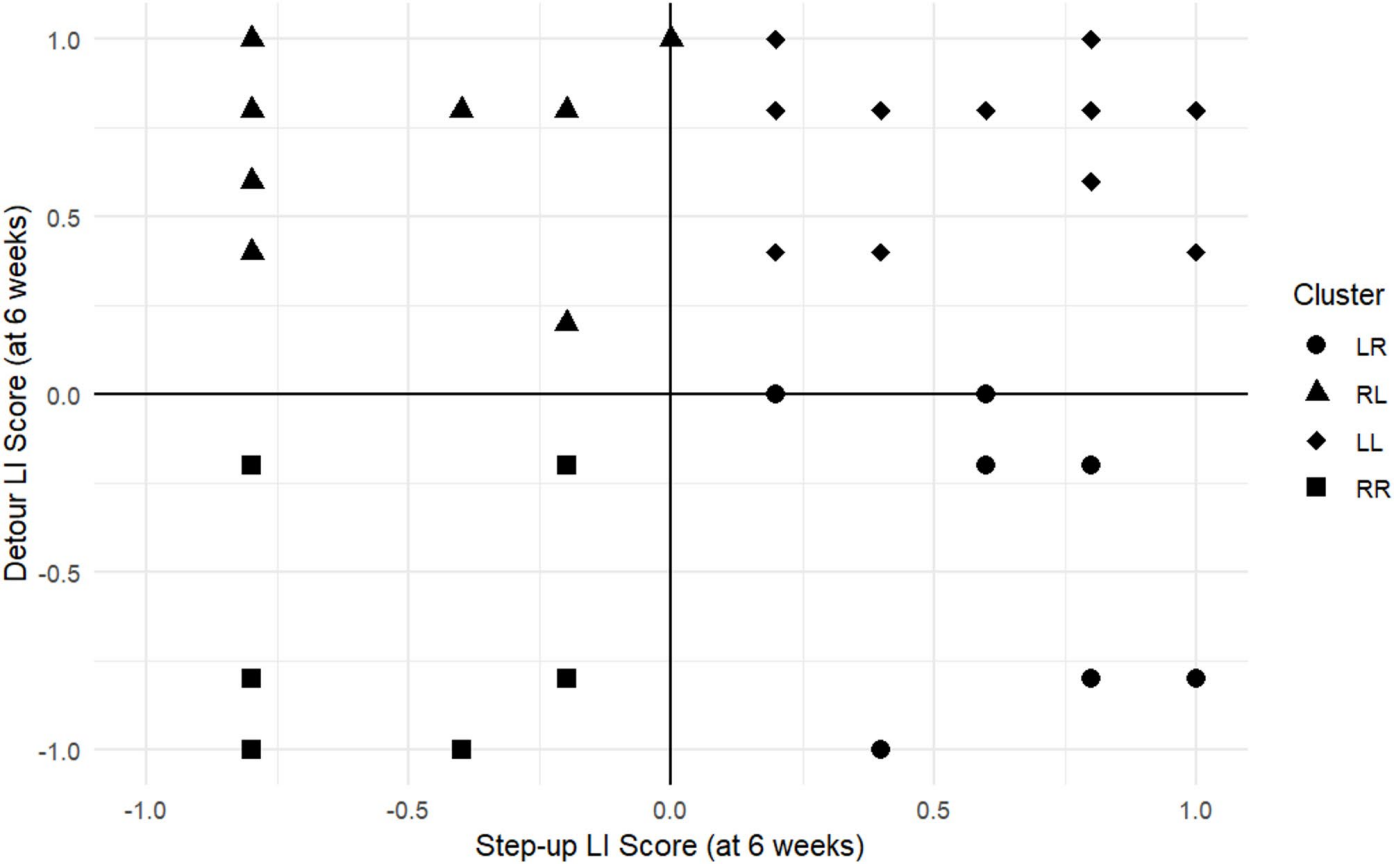
Graphical representation of the cluster analysis combining the step-up and detour directional laterality (LI) scores at 6 weeks of age. The key indicates the clusters of pigs who displayed a consistent lateral bias direction across both tasks (i.e., LL or RR), and the clusters of pigs who were inconsistent in the direction of their lateral preferences across the tasks (i.e., LR or RL).

Analysis identified four distinct, meaningful clusters that best represented the underlying data structure. These included a cluster for animals that were left-biased for the *step-up* task and right-biased for the *detour* task [LR]; right-biased for the *step-up* task and left-biased for the *detour* task [RL]; left-biased for both tasks [LL]) and right-biased for both tasks [RR]. Figure 9 shows the graphical representation of these clusters. The validation result, using the average Silhouette width score of the four clusters (SWS = 0.53, *n* = 50), indicated fairly well-defined clusters with reasonably good separation and cohesion. The clustering results align well with the LI scores of the two tasks, providing confidence in the analysis.

A chi-squared test revealed no significant difference in the number of pigs in each of the four clusters (χ^2^ = 7.12, df = 3, *p* = 0.07). There was no significant difference in the number of lateralised (*n* = 31, 62%) and ambilateral (*n* = 19, 38%) pigs for the cluster laterality (*p* = 0.12, binomial test). Lateralised pigs were no more likely to be left- (*n* = 20) than right-sided (*n* = 11) for this combined classification (*p* = 0.15, binomial test).

Mean LI and ABSLI scores for the *step up* and *detour* task at 6 weeks were calculated to establish the animals’ combined direction and strength of laterality. These scores deviated significantly from normality (*W* (50) = 0.911, *p* < 0.001), with animals veering more towards left-side preferences (mean combined LI score = 0.18 +/- 0.08; mean combined ABSLI scores = 0.51 +/-0.04). Male and female pigs did not differ significantly in either the direction (*U* = 295.00, *p* = 0.741) or strength (*U* = 282.00, *p* = 0.56) of their combined laterality scores.

## Discussion

This study explored side preferences in the domestic pig across a range of measures, including expressions of naturalistic behaviour (*lying side*), performance on a number of well-established tasks (*snout use*,* detour*,* step-up*) and *tail curling*. Findings showed that pigs had a significant side bias on the *detour* task when tested at 6 weeks of age, pointing to lateralisation at the level of the individual for this measure. There was no evidence of lateralised side use for any of the other measures and no significant sex differences. Findings revealed a lack of consistency in pigs’ side preferences between tasks, although there was stability in performance over time. The results raise questions as to the value of using certain measures of motor bias as an indicator of cerebral asymmetry in this species, particularly in the context of the working farm.

### Individual vs. population level laterality

#### Distribution of side preferences

Analysis revealed a roughly equal distribution of lateralised (51.5%) and non-lateralised (48.5%) pigs across the measures used in this study, although the proportion of animals showing left, right or ambilateral responses differed between measures.

*Lying side* was the most likely measure to yield ambilaterality, a finding that concurs with some research on other species. For example, McDowell et al.^44^ found no lateral bias for lying side in pet cats (although see^59^. Cows have also been reported to be generally ambilateral for this outcome^60,61^. Lying side is a measure that is likely to be determined by a range of extrinsic factors (e.g., availability of space, positioning of other animals), particularly in farm animals living in close quarters; as such, it may not be an overly useful tool for assessing side preferences in such species. That said, pronounced changes in lying side are certainly worth paying attention to^62^. Siivonen and colleagues^63^, for example, found that cows with experimentally-induced mastitis spent less of their time lying on the mastitic side than before the intervention. Likewise, Medrano-Galarza and others^64^ found that cows with mastitis displayed a greater degree of lateralised lying side than control animals.

Somewhat surprisingly, *snout use* also yielded largely ambilateral responses. Goursot et al.^33^ found individual-level lateralisation for snout use on a task that involved male pigs manipulating a flap door. It is difficult to know whether differences in the nature of the experimental protocols, husbandry practices or animals employed as subjects (i.e., genetic differences), are responsible for the discrepancy of the findings of these two studies. Further work is recommended to explore snout laterality in pigs, particularly considering the importance of this appendage for navigating and interacting with the environment^65,66^.

The animals in the present study were no more likely to be side-preferent than ambilateral for the *step-up* task. Goursot and associates^33^ assessed both ‘foot up’ and ‘foot down’ use in pigs during a snout use challenge, with results pointing to ambilaterality for both measures. Other studies recording foot use as an indicator of laterality in animals have yielded variable results. For example, Tomkins et al.^67^ reported more side-preferent, than ambilateral, dogs on a step-down task, whilst Wells et al.^52^ found no significant difference in the proportion of dogs that were ambilateral vs. side-preferent on the same measure. Cats, by contrast, have been reported to be more side-preferent than ambilateral for measures of ‘step down’ and ‘step over’^44^.

*Tail curling* yielded no evidence of a side bias. Only one other study has explored this measure as an indicator of laterality in pigs, with the animals showing a right-sided bias at the level of the population^33^. It is hard to draw conclusions on whether or not tail curling is a reliable measure of laterality in pigs given the discrepancy in results between these two studies. Both of the studies involved tail-docked (or tipped) animals, but differed in several methodological respects, e.g., number of data points (30^33^ vs. 10 [present investigation], sex of animals (male only^33^ vs. mixed [present investigation], presence of experimenter (inside pen^33^ vs. outside pen [present investigation]). Further work is certainly needed to explore tail laterality in pigs, perhaps in a population of intact animals, particularly considering the use of the tail in social communication^68^ and its links to emotional functioning and welfare^69^.

The majority of pigs in the current study showed a side preference on the *detour* task at 6 weeks of age. This type of challenge has been used extensively to assess lateral bias in species including fish, with many authors reporting strong side preferences on this measure^70–72^. A recent study, however, concluded that the detour task does not provide accurate, precise or repeatable estimates of motor bias in fish^73^. This does not appear to be the case in pigs, given the significant positive correlation in HI scores that emerged between repeated tests on this task. The role of learning must be considered. For example, Pongracz and colleagues^74^ found that dogs tested on a social learning detour task showed a preferred side consistent with the direction of their first successful trial. Although not recorded here, it is possible that the animals gained significant reinforcement for this task on their first trial, increasing their chances of repeat performance. It is still interesting, however, that the pigs in this investigation showed a directional preference. Of course, differences between the studies must be acknowledged, including species under scrutiny, the number of data points collected and the calculation of repeatability, as opposed to correlational, scores.

#### Direction and strength of side preferences

Plots of the animals’ LI scores revealed bimodal distributions of laterality for many of the measures recorded (*step-up*,* detour*,* tail curling*), pointing to a leaning towards side preferences on these outcomes at the level of the individual.

The strength of the pigs’ side preferences was found to be measure-specific, with stronger patterns of side use more evident for *tail curling* and *detour* than *snout use* and *lying side*. This is of interest when considered in relation to the ‘task complexity hypothesis’^75^, which suggests that more complex tasks typically elicit stronger lateralised biases. In this study, the *detour* and *snout-use* tasks were considered more demanding and cognitively challenging than measures of *tail curling* and *lying side*. Therefore, the observed results do not fully align with the hypothesis, as it would be expected that *snout use* would exhibit stronger patterns of lateralisation, and *tail curling* would show weaker patterns of lateralisation. Related to this, Keerthipriya et al.^76^ put forward their ‘organ complexity hypothesis’, arguing that strength of laterality differs between organs, with paired organs (e.g., forelimbs) being less complex than unpaired ones (e.g., elephant’s trunk, langur’s tail). Given that pigs use their snouts extensively^65^, one could consider this appendage to be an unpaired organ capable of performing complex tasks^33^. One might therefore have expected the strongest patterns of laterality on the *snout use* challenge, arguably the most complex task and one that involved an unpaired organ. It would be interesting to explore whether these weaker signs of lateral bias on this particular snout task are a simple feature of the experimental design or whether snout use simply serves no value as an indicator of cerebral asymmetry.

### Test repeatability

This study investigated the consistency of pigs’ side preferences over time, with lateral bias tasks (*snout use*,* step-up*,* detour*) undertaken at 3–4 weeks of age in the farrowing house repeated at 6–7 weeks of age in the weaner house. Findings pointed to good test-retest reliability, with animals demonstrating a positive correlation in the direction (although not strength) of their lateral biases for all three measures. This hints at stability in pigs’ side preferences over time. It must be noted that only a matter of weeks separated the test-retest of the subject animals (although these weeks spanned a large transition [before and after weaning]); whether pigs tested at a later point in time would show similar patterns of lateral bias remains unknown, although seems likely when considered in relation to other species. For example, primates develop a lateral preference during the infant-juvenile period of development that stretches through to adulthood^77–79^, domestic cats demonstrate consistency in the direction of their paw use between 6 and 12 months of age^47^, and dogs show stability in the direction and strength of their lateral preferences when tested twice on the same motor bias measures^18,52,80^. Interestingly, cats have been reported to display stronger paw preferences at adulthood than at both 12 weeks and 6 months of age^47^. Similar findings have been reported from both longitudinal^77–79^ and cross-sectional^81–83^ studies on primates. Many factors may influence the development of laterality, both at an environmental and genetic level^35^. As yet it is unknown to what extent lateralisation is influenced by, for example, hormones, birth order, learning or reinforcement effects, not to mention interactions with conspecifics and humans. Testing pigs at a later age, albeit not without its challenges from a logistical perspective, could yield some interesting data on the stability of lateral biases over the lifespan and the factors that might influence the ontogeny of lateralisation in this species. This would also be of value from an animal welfare perspective, allowing us to determine if there is an optimum time to assess side preferences in pigs, the factors that serve as stressors to animals in this context, and the types of pigs (e.g., those with a certain personality type, see^34^ that might be better able to cope with the key milestones and environmental challenges that occur along the way.

### Sex differences in laterality

This study explored, for the first time, sex differences in pigs’ side preferences. Male and female animals showed no significant difference in either the strength or direction of their side preferences for most of the tasks. This is somewhat surprising, as several studies have found a population split in directional laterality in certain species (e.g., primates^36–38^; horses^39,40^; dogs^41–43^; cats^44–47^, with male animals showing a preference for left limb use and females leaning more heavily towards right limb bias. *Tail curling* was the only measure that hinted at a sex difference in lateral bias, with females showing stronger, and more left-sided, biases than males; after correcting for multiple comparisons, however, these significant sex effects were negated. As mentioned earlier, Goursot et al.^33^ measured the tail curling direction of male pigs and found a significant leaning towards right-sidedness; this aligns with what was observed in our sample of male animals.

### Associations and patterns of laterality

Evaluating an animal’s lateral preferences across various tasks, rather than solely within a single task, is crucial for understanding its hemispheric dominance patterns, i.e., identifying whether there is a consistent left or right hemispheric dominance^16,33^. Authors have recognised that due to the multidimensional nature of laterality, left or right hemispheric dominance does not necessarily result in consistency of lateral preference across every lateral bias measure^84^. Individual, task-specific, and functional differences can influence the laterality outcome^52,75,76^. Nevertheless, identifying and utilising measures able to accurately elicit individuals’ lateral preferences for a given function remains of fundamental importance. Once established, examining laterality patterns across tasks at the individual level allows for overall assessment of their hemispheric dominance, comparable to McGrew and Marchant’s^86^ position on ‘true laterality’. This assessment of hemispheric dominance provides an indication of how an individual processes information at a cerebral level, which subsequently influences their behaviour and distinguishes them from others with different hemispheric dominance patterns^86^.

The present study yielded little in the way of significant correlations in either the strength or direction of the pigs’ side preferences between tasks. To further explore patterns of laterality across measures, a cluster analysis combining lateral preferences from *detour* and *step-up* tasks was carried out. Measures with a bimodal distribution were selected, as this suggested they were effective in establishing side preferences in pigs^33^. *Tail curling*, however, was excluded despite its bimodality. The shorter tail length observed in the animals in this study may not necessarily have reflected the direction the full-length tail would have curled, raising the question of whether tail curling in docked pigs is a reliable measure of laterality. This is an interesting observation, given that this study aimed to explore what tools could be useful for assessing lateral bias in the context of the working farm, where some animals will indeed have docked tails. Further week is needed to determine how tail curling in docked pigs compares to that of undocked animals, but as a result of the challenges presented here, it was deemed inappropriate to include this measure in the combined laterality analysis.

As in Goursot et al.’s^33^ work, the cluster analysis identified pigs with LR and RL biases, indicating opposing lateral preferences across measures. Whilst these results should be interpreted with caution due to the small number of measures included in the combined assessment, they suggest that these pigs may not have a tendency towards a dominant hemisphere. In contrast, the RR and LL clusters identified pigs with consistent lateral preferences across tasks, implying a preference for one hemisphere over the other. Previous research has indicated that hemispheric dominance can influence an animal’s susceptibility to welfare outcomes, with right hemisphere dominance associated with adverse welfare effects and left hemisphere dominance linked to positive welfare outcomes^7,16,20,87,88^.

## Limitations

Whilst this study used a relatively large number of laterality measures, only 10 data points per measure were recorded, largely in an effort to allow for greater efficiency of data collection in the context of the busy working farm. The appropriate number of data points required for the assessment of lateral bias has raised discussion^89^. Studies on animal laterality vary widely in the number of data points collected, from as few as 1^91^ to as many as 500^92^. Importantly, 10 data points, as collected in the current study, exceeds the minimum number (*n* = 6) required for binomial tests^92^, although it is still a factor to be considered.

## Conclusions

Overall, the results from this study suggest that lateralised behaviour in the domestic pig is specific to the individual, rather than the group, and is not overly consistent between tasks. It is, however, stable over time, at least in the short-term. The *step-up* and *detour* tasks were considered more useful indicators of lateral bias in this species than either *lying side* or *snout use* and could be easily used in the context of the working farm. It was unclear whether *tail curling*, included deliberately in case it harboured any merit as an easy to implement measure, yielded reliable information in this population of docked animals. The study provided little evidence of any sex differences in laterality in pigs. What is still to be determined, and is currently under analysis, is whether or not pigs’ lateral biases are correlated with any animal welfare measures. This information will hopefully allow us to establish whether such measures hold any merit as a non-invasive tool for assessing porcine welfare risk.

## Supplementary Information

Below is the link to the electronic supplementary material.

Supplementary Material 1

## Data Availability

The dataset generated during the current study is included in the Supplementary Information files.
